# Clinical diagnosis and mutation analysis of four Chinese families with succinic semialdehyde dehydrogenase deficiency

**DOI:** 10.1186/s12881-019-0821-z

**Published:** 2019-05-22

**Authors:** Ping Wang, Fengying Cai, Lirong Cao, Yizheng Wang, Qianqian Zou, Peng Zhao, Chao Wang, Yuqin Zhang, Chunquan Cai, Jianbo Shu

**Affiliations:** 1 Tianjin Pediatric Research Institute, Tianjin Children’s Hospital, No.238, Longyan Road, Beichen District, Tianjin, 300134 People’s Republic of China; 2 Department of Physiology, Tianjin Medical College, Tianjin, 300222 China; 3 Graduate College of Tianjin Medical University, Tianjin, 300070 China; 4 Department of Rehabilitation, Tianjin Children’s Hospital, Tianjin, 300134 China; 5 Department of Neurology, Tianjin Children’s Hospital, Tianjin, 300134 China; 6 Department of Neurosurgery, Tianjin Children’s Hospital, No.238, Longyan Road, Beichen District, Tianjin, 300134 People’s Republic of China

**Keywords:** Succinic semialdehyde dehydrogenase deficiency, *ALDH5A1* gene, Multidimensional analysis, Novel mutation

## Abstract

**Background:**

Succinic semialdehyde dehydrogenase (SSADH) deficiency is a rare autosomal recessively-inherited defect of γ-aminobutyric acid (GABA) metabolism. The absence of SSADH, which is encoded by aldehyde dehydrogenase family 5 member A1 (*ALDH5A1*) gene, leads to the accumulation of GABA and γ-hydroxybutyric acid (GHB). Few cases with SSADH deficiency were reported in China.

**Case presentation:**

In this study, four Chinese patients were diagnosed with SSADH deficiency in Tianjin Children’s Hospital. We conducted a multidimensional analysis with magnetic resonance imaging (MRI) of the head, semi quantitative detection of urine organic acid using gas chromatography-mass spectrometry, and analysis of *ALDH5A1* gene mutations.

Two of the patients were admitted to the hospital due to convulsions, and all patients were associated with developmental delay. Cerebral MRI showed symmetrical hyperintense signal of bilateral globus pallidus and basal ganglia in patient 1; hyperintensity of bilateral frontal-parietal lobe, widened ventricle and sulci in patient 2; and widened ventricle and sulci in patient 4. Electroencephalogram (EEG) revealed the background activity of epilepsy in patient 1 and the disappearance of sleep spindle in patient 2. Urine organic acid analysis revealed elevated GHB in all the patients. Mutational analysis, which was performed by sequencing the 10 exons and flanking the intronic regions of *ALDH5A1* gene for all the patients, revealed mutations at five sites. Two cases had homozygous mutations with c.1529C > T and c.800 T > G respectively, whereas the remaining two had different compound heterozygous mutations including c.527G > A/c.691G > A and c.1344-2delA/c.1529C > T. Although these four mutations have been described previously, the homozygous mutation of c.800 T > G in *ALDH5A1* gene is a novel discovery.

**Conclusion:**

SSADH deficiency is diagnosed based on the elevated GHB and 4, 5DHHA by urinary organic acid analysis. We describe a novel mutation p.V267G (c.800 T > G) located in the NAD binding domain, which is possibly crucial for this disease’s severity. Our study expands the mutation spectrum of *ALDH5A1* and highlights the importance of molecular genetic evaluation in patients with SSADH deficiency.

## Background

Succinic semialdehyde dehydrogenase (SSADH, OMIM: 271980) deficiency, also known as 4-hydroxybutyric aciduria, is a rare autosomal recessive genetic disorder that affects the degradation of γ-aminobutyric acid [1]. SSADH catalyzes succinic semialdehyde (SSA) to succinic acid (SA) in the final step of γ-aminobutyric acid (GABA) catabolism. In the absence of SSADH, SSA is converted into γ-hydroxybutyric acid (GHB) and other related metabolites. GHB can be converted to 3-deoxytetronic acid (3DT) or 2-deoxytetronic acid (2DT) via α or β oxidation. GABA and GHB accumulate in the brain, blood, urine, and cerebral spinal fluid. The clinical features of this disease are various and non-specific, including developmental delay, intellectual deficit, hypotonia, ataxia and seizures, and especially language delays [2–4]. Severity ranges from mild impairment to a relentlessly progressive neurodegenerative course with intractable seizures in infancy [5]. GABA_A_ receptor down regulation was previously reported as one of the pathophysiological mechanisms [2], but current data reveal a surprisingly complex pattern of pathophysiology including oxidative stress parameters and dysregulation of autophagy and mitophagy [1].

The gene of *ALDH5A1* with a full length of 1605 bp containing 10 exons, located in chromosome 6p22.3, and codes 535 amino acids with the first 47 residues representing the mitochondrial targeting peptide [4, 6]. The monomeric SSADH consisted of three domains: an oligomerization domain (174–195 and 525–535), a catalytic domain (308–508), and an N-terminal NAD-binding domain (residues 48–173, 196–307, and 509–524) that is responsible for the binding of NAD^+^ to the enzyme [7]. To date, more than 56 mutations of *ALDH5A1* have been identified [8] and are considered as the main cause for the decrease or elimination of SSADH activity.

Only a few cases of SSADH deficiency have been reported in mainland China [9–12]. In this study, we summarized the characteristics of clinical data and analyzed *ALDH5A1* gene mutations in four Chinese patients with SSADH deficiency diagnosed in our hospital. We present a novel mutation (c.800 T > G) in *ALDH5A1*, which enriches the analysis of *ALDH5A1* mutation spectrum. Based on our correlation analysis between SSADH protein function and the clinical data of patients, we speculate that the mutation in NAD binding domain (p.S510F and p.V267G) is crucial for this disease’s severity.

## Case presentation

Four Chinese patients (two males and two females) at the age of 86 days to 5 years were diagnosed with SSADH deficiency in Tianjin Children’s Hospital in China from June 2012 to September 2016. These patients had no family history. Detailed data are shown in Table 1. Cerebral magnetic resonance imaging (MRI), electroencephalogram (EEG), and routine tests were conducted. Semi-quantitative analysis of organic acids in urine was performed by gas chromatography-mass spectrometry (GC/MS), and the results were included in Table 2. Genetic analysis was performed using blood samples collected from the patients and their parents. One hundred healthy children were enrolled as the control group. DNA was extracted by gDNA Mini Kit (BIOMIGA, USA) according to the manufacturer’s instructions. PCR amplification for 10 exons and flanking intronic regions of *ALDH5A1* gene was performed, whose primers and reaction condition were designed based on relevant references [12]. PCR products were electrophoresed on 2% agarose gels and sequenced by biological company (GENEWIZ, Tianjin). The sequencing results were compared with the reference sequence of *ALDH5A1* (NM_170740) of NCBI.

**Table 1:** Clinical data of 4 Chinese patients affected with SSADH deficiency

Patients	1	2	3	4
Gender	F	M	M	F
Age of diagnosis	3 y	86 d	5 y	8 m
Cesarean delivery	Y	Y	N	N
Family history	N	N	N	N
Consanguineous	N	N	Y	N
Convulsions	Y	Y	N	N
Developmental delay	Y	Y	Y	Y
Hypotonia	N	N	N	Y
Hyporeflexia	N	N	N	Y
Sleep disturbances	N	Y	N	Y
Abnormal EEG	Y	Y	UN	N
Abnormal MRI	Y	Y	UN	Y
Metabolic acidosis	N	Y	N	N
4-HB urine	Y	Y	Y	Y

*F* female, *M* male, *y* years, *d* days, *m* months, *N* no, *Y* yes, *UN* unknown

**Table 2:** Analysis of organic acid in urine by GC/MS

Metabolites	Case1	Case2	Case 3	Case 4	Reference range
4-hydroxybutyric acid	1.533	4.887	2.178	9.603	< 0.001
3,4-dihydroxybutanoic acid	1.695	9.073	1.796	16.173	< 0.895
2,4-dihydroxybutanoic acid	0.549	1.425	0.788	8.216	< 0.208
4,5-dihydroxyhexanoic acid	0.958	6.320	1.548	4.197	< 0.001

Value: metabolite peak area ratio to creatinine

The alignment for amino acid sequence of SSADH in Human, Chimpanzee, Bos, Rattus, Anser, Xenopus, and Zebrafish were conducted by DNAMAN software. The functional prediction of gene mutation was performed by Polyphen2 (http://genetics.bwh.harvard.edu/pph2/) and SIFT (http://sift.jcvi.org/).

### Patient 1

Patient 1 was a 3-year-old female born at term by cesarean delivery with the history of aspiration of amniotic fluid and was not awaked easily for one day after birth. She was admitted to the hospital due to cough for 5 days and intermittent convulsions for 3 days. She had partial convulsions without fever while awake, she slept after remission, most convulsions were less than two minutes in duration. She had no movement or language disorders during the postictal period. She also presented with developmental delay, especially language delay. Cerebral MRI displayed symmetrical hyperintensity of bilateral basal ganglia and globus pallidus on T2-weighted image (T2WI) and hyperintensity of the bilateral parietal lobe white matter on fluid-attenuated inversion recovery (FLAIR) imaging. EEG revealed high voltage of delta and theta wave at approximately 2–4 Hz. Urinary organic acid analysis showed elevated GHB concentrations.

Molecular analysis of *ALDH5A1* confirmed a compound heterozygous mutation of c.527G > A/c.691G > A (p.G176E/p.E231K) in exons 3 and 4. c.527G > A was inherited from her mother, whereas c.691G > A was inherited from her father. We have previously reported these two mutations [8, 12]. Bioinformatics analysis indicated that p.G176 and p.E231 are highly conserved among species. These mutations were predicted to be “Probably damaging” and “Possibly damaging” based on the Polyphen2 software**.**

### Patient 2

Patient 2 is an 86-day-old male conceived through in vitro fertilization and born at 37 weeks by cesarean section due to cord around the neck. His mother had an abnormal reproductive history. Her first baby died of convulsion at 23 days after birth, while her second pregnancy was ectopic. This patient was referred to the hospital due to intermittent convulsions for more than 2 months and exacerbation for 2 days. Partial convulsions occurred while he was awake or asleep without fever. He was back to normal without movement disorders. He presented with decreased attention, poor head control, and thumb abduction. Cerebral MRI revealed patchy hyperintensity in bilateral frontal and parietal lobe on T2WI. Widened ventricle and the interval outside the cerebral were also noted in the cerebral MRI. EEG showed sleep spindle asynchrony. Blood gas analysis indicated metabolic acidosis. GHB in urine was significantly elevated according to urinary organic acid analysis. Unfortunately, this patient died of pneumonia despite rescue efforts.

A homozygous mutation variant, c.1529C > T (p.S510F), was identified in exon 10 of the *ALDH5A1* gene in this patient. Subsequent targeted mutational analysis of exon 10 of his mother confirmed the segregation of the variant. The healthy father did not carry the sequence variant. p.S510 is highly conserved, its mutation is predicted to be “Probably damaging.”

### Patient 3

Patient 3 was a 5-year-old boy born at full term with the history of hypoxia after birth. He was admitted to the hospital due to developmental delay complicated with lack of sleep. Urinary organic acid analysis showed a high level of GHB. EEG and cerebral MRI were unclear because both were performed by other hospitals.

This patient harbored a novel homozygous mutation of c.800 T > G (p.V267G) in exon 5, which he inherited from his parents. Bioinformatics analysis revealed that p.V267 is highly conserved among species. The mutation was predicted to be “probably damaging” and “disease causing” with a score of approximately equal to 1**.**

### Patient 4

**Fig. 1:**
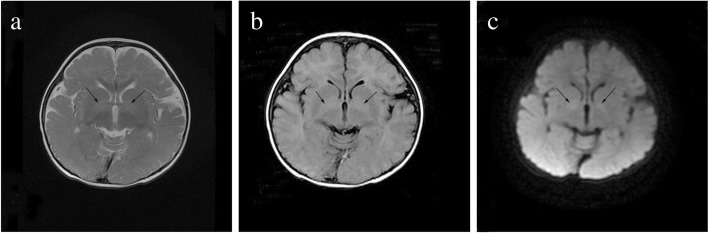
Cerebral MRI of patient 4 displayed hyperintense signal of bilateral globuspallidus (arrow), widened ventricle, and interval outside the cerebral on T2WI (**a**), FLAIR (**b**) and diffusion weighted image (**c**)

Patient 4 was an 8-month-old girl born at full term by natural delivery and referred to the hospital because of developmental delay. The clinical manifestations showed slow pupillary light reflex and hyporeflexia and hypotonia of all limbs. Cerebral MRI revealed widened ventricle, cistern, and sulci, which was considered as delayed myelination (Fig.1). EEG was normal. Urinary organic acid analysis showed an increased excretion of GHB.

A compound heterozygous mutation of c.1344-2delA/c.1529C > T was identified in exons 9 and 10 in this girl. The mutation c.1344-2delA located in the intron splice site, and p.S510F (c.1529C > T) belongs to the NAD-binding domain. These mutations are separately inherited from both of her parents.

## Discussion and conclusion

SSADH deficiency is a rare inherited metabolic disease characterized by the metabolic disorder of GABA. The incidence rate is unknown but has been estimated at 1:100000 [1]. The first case was reported by Jakobs in 1981 [13]_._ Parental consanguinity has been reported in approximately 40% of affected population [9]. The median age at diagnosis of SSADH is 2 years old, but underdiagnosis is suspected [14]. Nearly 80% of patients are diagnosed at the age of 5 years, although a few ones are confirmed in late childhood or adolescence [14], and sporadic patients are diagnosed during adulthood [15]. In this study, patient 3 was born to consanguineous parents. These four Chinese patients were diagnosed at the age range of 86 days to 5 years. The signs and symptoms of SSADH deficiency are often non-specific, including developmental delay, hypotonia, hyporeflexia, ataxia, neuropsychiatric problems, and epilepsy. Hypotonia and neuropsychiatric problems each have occurred in 70% of patients, and approximately half of these patients have epilepsy [16]. Neuropsychiatric symptoms, involving disabling obsessive compulsive disorder and anxiety in addition to inattention, hyperactive behavior, and sleep disturbances, were also noted [17]. EEG shows generalized spike-waves, photosensitivity discharge, and sleep spindle asynchrony [18]. In this study, patients 1 and 2 presented with convulsions, and all the patients had the history of developmental delay. Patient 4 displayed hypotonia and hyporeflexia. Patient 2 and 4 exhibited decreased attention and sleep disturbances, respectively. EEG showed the background of epilepsy in patient 1 and the disappearance of sleep spindle in patient 2. The clinical manifestations of these patients are consistent with the signs and symptoms of SSADH deficiency.

In this study, we identified four patients with different *ALDH5A1* gene mutations. The c.800 T > G (p.V267G) mutation in patient 3 is a novel mutation. Bioinformatics analysis revealed that this mutation was highly conserved across species and might play an important biological role. This mutation was predicted to be “probably damaging” and “disease causing” based on the Polyphen2 software with a score of approximately equal to 1. 267 V is located in the N-terminal NAD-binding domain, the mutation in this domain may influence the combination of SSADH with the NAD^+^ molecule.

SSADH deficiency is diagnosed based on the elevated GHB and 4, 5DHHA by urinary organic acid analysis. SSADH activity or molecular genetic analysis of *ALDH5A1* gene are used to confirm this disorder [4]. In this study, the GHB in urine was elevated dramatically in these four Chinese patients. Genetic investigation also identified five mutations of *ALDH5A1* gene. Cerebral MRI showed symmetrical hyperintense signal of bilateral globus pallidus and basal ganglia in patient 1; hyperintensity of bilateral frontal-parietal lobe, widened ventricle, and sulci in patient 2; and widened ventricle and sulci in patient 4.

In conclusion, we reported five *ALDH5A1* gene mutations in four Chinese children with SSADH deficiency. Among these mutations, one novel mutation is presented uniquely in our study. Our study may provide useful information for the gene mutation analysis of *ALDH5A1* and contribute to the diagnosis of SSADH deficiency in clinical practices and in the screening of *ALDH5A1* gene mutation carriers, especially those undergoing neonatal screening with family histories.

## Abbreviations

4,5DHHA: 4,5-dihydroxyhexanoic acid
ALDH5A1: Aldehyde dehydrogenase family 5 member A1
FLAIR imaging: Fluid-attenuated inversion recovery imaging
GABA: Gamma-aminobutyric acid
GC/MS: Gas chromatography-mass spectrometry
GHB: γ-hydroxybutyric acid
MRI: Magnetic resonance imaging
SNPs: Single nucleotide polymorphisms
SSADH: Succinic semialdehyde dehydrogenase
